# A model of classroom social climate, foreign language enjoyment, and student engagement among English as a foreign language learners

**DOI:** 10.3389/fpsyg.2022.933842

**Published:** 2022-08-17

**Authors:** Hamed Mohammad Hosseini, Jalil Fathi, Ali Derakhshesh, Sepideh Mehraein

**Affiliations:** ^1^Department of English Language and Literature, Faculty of Language and Literature, Islamic Azad University, Tehran, Iran; ^2^Department of English and Linguistics, Faculty of Language and Literature, University of Kurdistan, Sanandaj, Iran; ^3^Department of English Language and Literature, Faculty of Letters and Human Sciences, Shahid Beheshti University, Tehran, Iran; ^4^Department of English Language and Literature, Faculty of Foreign Languages and Literatures, University of Tehran, Tehran, Iran

**Keywords:** classroom social climate, foreign language enjoyment, student engagement, SEM, EFL learners

## Abstract

With the advent of positive psychology in second language (L2) learning, some researchers have undertaken empirical studies to explore emotional variables affecting L2 learning and how positive emotions can enhance the engagement of L2 learners. As an attempt to contribute to this research domain, this project sought to test a model of student engagement based on classroom social climate (CSC) and foreign language enjoyment (FLE) among English language learners in Iran. A sample of 386 intermediate English as a foreign language (EFL) learners took part in this survey by completing the online battery of questionnaires. Structural equation modeling (SEM) was employed for the analysis of the gathered data. The results showed that both CSC and FLE were significant predictors of student engagement, with FLE acting as a stronger predictor. Furthermore, CSC exerted a slight influence on FLE. The findings of the present study verify the contributions of positive psychology to L2 pedagogy, implying that pleasant perceptions of learning context and positive emotions can lead to further student engagement.

## Introduction

In the context of L2 learning, one cannot think of successful learners without having at least some form of student engagement. This construct, which is conceptualized as students’ active involvement in their learning activities and their commitment to learning goals (Christenson et al., 2012; Aparicio et al., 2021), can help learners achieve their learning outcomes and sustain educational improvement. Engaged students have some degree of participation and persistency in the learning process, exert efforts in classroom tasks, and exhibit different levels of interest, motivation, and enjoyment to learn (Fredricks et al., 2004; Derakhshan et al., 2022a). Conversely, non-engaged learners are bored learners who feel apprehension or sometimes angry about participating in the classroom and perform poorly in a variety of subjects (Kelly, 2008; Mantzios and Egan, 2019). As such, successful learning is dependent on the degree to which learners are involved in learning tasks (Wang and Pomerantz, 2009). In the last two decades, student engagement and its various functions have received an increasing amount of attention (Shernoff and Schmidt, 2008; Christenson et al., 2012; Ryu and Lombardi, 2015; Bond, 2020; Bowden et al., 2021). Research has demonstrated that this multidimensional construct is highly dynamic and context-dependent (Goldin et al., 2011) with different levels of ecological influence (Christenson et al., 2012). More recently, empirical studies have also explored student engagement in connection with learner-related variables such as autonomy, self-efficacy, competence, and relatedness (Oga-Baldwin and Nakata, 2017; Mercer, 2019; Noels et al., 2020). However, our understanding of how classroom social climate (CSC), as a context-dependent construct, and foreign language enjoyment (FLE), as a positive internal variable, are related to student engagement is still putative.

The social-ecological characteristics of the EFL classroom, referred to as classroom climate, are a combination of teachers’ and learners’ feelings in the classroom which emanate from their behavior. Teacher behavior includes teachers’ communication style, classroom management, expressing interest in student ideas, and offering help to students, while student behavior includes continuous involvement in activities, cooperation, and competition (Garrett, 2008; Schiller, 2009; Brandmiller et al., 2020; Yang et al., 2020). Also, research suggests that a positive climate in the class can promote student learning motivation (Peng, 2012; Joe et al., 2017), self-efficacy (Lim and Fraser, 2018), achievement (Gedamu and Siyawik, 2015), attitude (Khajavy et al., 2016), and willingness to communicate (Khajavy et al., 2018; Zhang et al., 2022). Motivated by the emerging field of positive psychology, FLE is defined as the perception of pleasure provided by the L2 learning classroom (Lee, 2020). Learners who experience enjoyment can envision goals, solve problems creatively, and regulate their learning behavior properly (Pekrun and Linnenbrink-Garcia, 2014). Enjoyment in language learning is likely to be triggered by teachers (e.g., pedagogical practice and supportive attitudes toward students), learners (e.g., sense of accomplishment and excellent performance), and atmosphere (e.g., group activities, classroom climate) (Dewaele and Dewaele, 2017).

In spite of some recent attention directed to FLE, this construct is still under-explored and merits further empirical investigations. Although FLE, CSC, and student engagement have been investigated over recent years, the simultaneous associations among these three constructs in EFL contexts seem to be absent in the related literature. To further enrich the literature in this area, this study uses survey data from Iranian English language learners to test a model hypothesizing CSC and FLE as predictors of student engagement. Examining this model offers a helpful account of student engagement in the EFL context. Furthermore, the hypothesized model can aid L2 learners in understanding what variables influence their engagement in English. Accordingly, they can manipulate resources at their disposal to become optimally engaged with their learning.

## Literature review

### Student engagement

In recent years, SLA researchers have taken a comprehensive view of an array of emotions by incorporating positive emotions such as engagement, motivation, and enjoyment, which are essential to L2 learning (Seligman, 2018). There are good reasons to believe that studying positive emotions will improve our understanding of the language learning process: they assist learners to ameliorate the adverse effects of negative emotions, facilitate exploration and playfulness which in turn results in more effective learning through creating a social bond, and help build resiliency in the face of difficulty (Dewaele and Macintyre, 2014; Dörnyei and Ryan, 2015; MacIntyre et al., 2016). Among the positive emotions, student engagement, which accounts for the E construct of the PERMA framework, is considered a significant booster for academic achievement as well as one of the cornerstones of positive psychology (Seligman, 2011). Therefore, this construct has been brought under investigation in the field of SLA for over 70 years (e.g., Pekrun and Linnenbrink-Garcia, 2012; Mercer, 2019; Mercer and Dörnyei, 2020) with the meaning of the construct evolving over time.

There are also different definitions for the student engagement construct and it can take many forms. However, to better understand the construct of student engagement and to resolve terminological confusion, Svalberg (2009) made a distinction between engagement and other similar constructs such as involvement, motivation, and commitment. She argued that each of these neighboring constructs can function as a sub-construct covering only part of the engagement meta-construct. Thus, an increasing amount of research has examined student engagement as a pivotal factor in language learning and scrutinized its impact on student learning and institutional effectiveness. It is widely agreed that student engagement is a multidimensional and complex construct. One view is that student engagement comprises three core dimensions: behavioral, emotional, and cognitive (Fredricks et al., 2004). In this framework, the first dimension refers to how consistent the student is in terms of effort, participation, attention, homework, and other desired behaviors in the class contexts. Emotional engagement can be related to the presence of students’ positive and negative emotions such as interest, boredom, or anxiety during task involvement. Finally, the cognitive dimension refers to students’ depth of processing and their use of metacognitive strategies. Later, a fourth dimension, social engagement, was added to the first three components (Svalberg, 2009; Philp and Duchesne, 2016); however, this new dimension could not be considered a required aspect since all the other dimensions are socially situated (Mercer and Dörnyei, 2020). As an alternative, agentic engagement, which refers to the “students’ constructive contribution into the flow of the instruction they receive,” was suggested (Reeve and Tseng, 2011, p. 258). The four dimensions of behavioral, emotional, cognitive, and agentic accord with the learning processes of acting, feeling, thinking, and communicating, respectively (Reeve, 2013; Wang and Eccles, 2013). Although past research has indicated that students’ behavioral, emotional, and cognitive engagements help students make progress (e.g., Finn and Zimmer, 2012; Reeve, 2013), more recent studies have demonstrated that agentic engagement is associated with greater achievement, autonomy, and motivation (Matos et al., 2018; Reeve and Shin, 2020).

Despite the amount of accumulated research on the components of student engagement, our understanding of the predictors of this construct is just developing. Many studies have investigated teacher-related factors such as teacher instrumental support, teacher obstruction, teacher instruction, and teacher motivation as predictors of student engagement (e.g., Demir, 2011; Scott and Hirn, 2014; Strati et al., 2017). For instance, Demir (2011) examined how teachers’ intrinsic and extrinsic motivation affect student engagement. The results demonstrated that both types of motivation significantly predicted student engagement, with intrinsic motivation as a stronger predictor. Moreover, the predicting roles of context-related factors such as academic environment and school climate (e.g., Karabchuk and Roshchina, 2022) have been explored. In their study, Karabchuk and Roshchina (2022) explored the role of the academic environment as well as students’ backgrounds as predictors of student engagement. Their results indicated that the academic environment is a strong predictor of student engagement. Another line of studies has examined the predictive role of learner-related aspects such as students’ self-esteem and self-efficacy (e.g., Walker et al., 2006; Shernoff et al., 2016). In their study, Walker et al. (2006) studied how three factors of identification with academics, motivation, and self-efficacy can predict cognitive engagement. The results of path analysis confirmed that each factor contributes to the prediction of cognitive engagement. Employing a mixed-methods study, Dewaele and Li (2021) explored the relationships among teacher enthusiasm, emotional constructs, such as boredom and enjoyment, as well as learning engagement among a big sample of participants in China. Results revealed significant associations among the constructs. Furthermore, enjoyment and boredom acted as mediators in the proposed model. Qualitative data clarified the causes of the inter-relations among these constructs.

### Classroom social climate

One of the important factors which influences what happens in the learning process is the social climate that reigns in the classroom. The quality, quantity, and direction of the relationship between teacher and learners and among learners which shapes learners’ motivation and performance can be seen as part of CSC (Fraser, 1986). In classes with a positive social climate, learners are more likely to expend effort, use self-regulated learning strategies, and seek help (Alzubaidi et al., 2016). Besides, learners are more cognitively engaged in such a productive climate and express their ideas more freely (Ryan and Patrick, 2001).

Attempts to unearth the dimensions of CSC have yielded four complementary constructs: *teacher academic support*, i.e., learners’ perception of the teacher’s personal support showing his concern about how much the students learn; *teacher emotional support*, i.e., learners’ perception that the teacher likes them as an individual and supports their wellbeing; *classroom mutual respect*, i.e., learners’ perception that the teacher encourages them to value each other’s feelings; and *task-related interaction*, i.e., learners’ perception that the teacher encourages them to interact with each other during the class (see Patrick et al., 2007, 2011). Underlying these dimensions and as demonstrated by research in this area (e.g., Fraser et al., 2010; Lerdpornkulrat et al., 2018) is the assumption that the larger the extent of these perceptions, the more expected the learners’ outcomes, namely, engagement, achievement, and motivation.

Classroom climate in the EFL context is fundamentally interpersonal in nature as it is established by the actions carried out among individuals, either as a teacher or a learner. A responsive social climate in the classroom may stimulate learners from diverse backgrounds to willingly engage in learning activities, hence increasing the probability of reaching their goals satisfactorily (Allodi, 2010). Regarding language learning CSC, previous research has predominantly focused on the association between CSC and various key variables (Derakhshan et al., 2022b). Waluyo and Tuan (2021), e.g., revealed that EFL learners’ help-seeking avoidance was not strongly associated with the social climate of the classroom and thus help-seeking was not predicted by CSC. They also found that social climate contributed significantly to the learners’ language proficiency. Studies have also demonstrated classroom environment, as an important contextual factor, directly or indirectly influences willingness to communicate (WTC). As an example, the results of Peng’s (2019) investigation showed that audio/video resources, as well as voice/facial expressions, exert a direct effect on CSC and that CSC is the strongest predictor of WTC. Also, in the study by Wang H. et al. (2021, p. 8) “an indirect effect was found between class social climate and L2WTC in class through both positive emotions (i.e., enjoyment and pride) and negative emotions (i.e., anxiety and boredom).” In terms of achievement, Liu and Fraser (2013) found that the relationship between learners’ perception of classroom climate and their academic achievement is mediated by their attitudes toward English.

In another study, Wei et al. (2019) investigated the impact of grit on L2 performance by examining the mediating role of FLE and the classroom environment. A sample of 832 middle school learners participated in this study. The findings revealed that grit had a positive effect on L2 performance. Additionally, FLE was a significant mediator affecting the interplay between grit and L2 performance, and the classroom environment influenced the association among grit, FLE, and L2 performance. The extant literature verifies the notable contribution of pleasant learning contexts to enhancing student engagement (e.g., Klem and Connell, 2004; Gest et al., 2005; Gauley, 2017; Del Toro and Wang, 2021; Hiver et al., 2021). Ryan and Patrick (2001) also found that students’ perception of their social classroom environment was correlated with their engagement and motivation. Using longitudinal design and collecting four waves of data, Del Toro and Wang (2021) found that cultural socialization within African American schools could substantially affect adolescents’ school engagement with the mediation of school climate. Reviews of research clearly show that (a) past CSC studies have less focused on the subject of language and that (b) these studies have rarely, if at all, investigated this positive emotion as the antecedent of engagement, both of which are catered for in our study.

### Foreign language enjoyment

FLE, as a positive emotion, is in agreement with the emergent domain of positive psychology and more precisely with broaden-and-build theory (Fredrickson, 2001) which postulates that an L2 learner can benefit from positive emotions as they “share the ability to broaden people’s momentary thought-action repertoires and build their enduring personal resources, ranging from physical and intellectual resources to social and psychological resources” (Fredrickson, 2001, p. 220). These resources are essential to an individual’s future prosperity (Fathi and Mohammaddokht, 2021). To fully understand enjoyment, a distinction should be made between enjoyment and pleasure. According to Nakamura and Csikszentmihalyi (2014), pleasure is the fulfillment of homeostatic needs like bodily comfort, sex, and hunger, whereas enjoyment is the feeling that people experience when they transcend the limits of homeostasis as well as stretching beyond oneself to accomplish something challenging. Enjoyment is conceptualized as “a positive state where challenges and skills to meet them are aligned well” (Dewaele and Macintyre, 2014, p. 242). Csikszentmihalyi (2004) considers enjoyment as an integral component of the concept of flow and defines it as a complex emotion that is experienced when learners’ skills come in line with the challenges that a task represents. Csikszentmihalyi (2004) elaborated how overwhelming activities which exceed learners’ current level of ability lead to distress and anxiety, while optimally challenging activities bring about feelings of flow and enjoyment.

Empirical studies on SLA-related enjoyment have pursued either the conceptualization and measurement of this positive emotion (e.g., Dewaele and Dewaele, 2017; Jin and Zhang, 2018; Botes et al., 2021; Wang X. et al., 2021), the potential links between FLE and socio-biographical variables (e.g., Jiang and Dewaele, 2019; Li et al., 2020), or the relationship between FLE and other psychological traits. FLE is conjectured to help learners attend to, process, and learn a target language more efficiently. FLE has been also empirically substantiated to affect learning outcomes (Dewaele and Macintyre, 2014; Jin and Zhang, 2018; Botes et al., 2020). Khajavy et al. (2018) found that enjoyment positively predicted learners’ willingness to communicate at both learner and classroom levels. Prior research has also shown that FLE is a significant predictor of actual L2 performance (Dewaele and Alfawzan, 2018; Saito et al., 2018). Employing Chinese EFL students, Li (2020) addressed the relationship between trait emotional intelligence (TEI), FLE, and learning achievement. The outcomes of mediation testing showed (1) the influence of FLE on (perceived/actual) achievement, (2) the impact of TEI on FLE, and (3) the mediating impact of FLE on the correlation between participants’ TEI and L2 learning outcomes. In a similar study, Li et al. (2020) used correlation regression analysis to test the role of a personal (i.e., TEI) and an environmental [i.e., classroom environment (CE)] factor in forming L2 emotions. They found that TEI and CE jointly and separately predicted foreign language anxiety and FLE significantly. The mediating role of FLE has been explored by some studies. For example, Wei et al. (2019) explored the influence of grit on foreign language performance (FLP) and found that not only does grit positively affect FLP but also their relationship is mediated by FLE. Also, in the context of Chinese universities, a study with third language learners confirmed the association between learners’ motivation and language proficiency is moderated by FLE (Zhang et al., 2020). Also, employing a mixed-methods study, Guo (2021) confirmed the reciprocal association between FLE and four underlying dimensions of student engagement among Chinese EFL learners. In addition, a favorable classroom environment has a lion’s share of variance in creating positive emotions in EFL contexts (Dewaele and Macintyre, 2014; Khajavy et al., 2018; Shao et al., 2019; Li et al., 2021).

## The hypothesized model

For the purpose of examining the interconnections among the constructs (i.e., FLE, CSC, and student engagement), a structural model is hypothesized. The specifications of the model are in line with the theoretical and empirical backgrounds of the constructs. Based on the conceptualization of FLE (Dewaele et al., 2018; Li et al., 2018), student engagement (Reeve, 2013; Reeve et al., 2020), and the findings of some studies (e.g., Klem and Connell, 2004; Shernoff et al., 2014; Dincer et al., 2019; Mercer and Dörnyei, 2020; Guo, 2021), FLE is hypothesized to influence student engagement.

**FIGURE 1:**
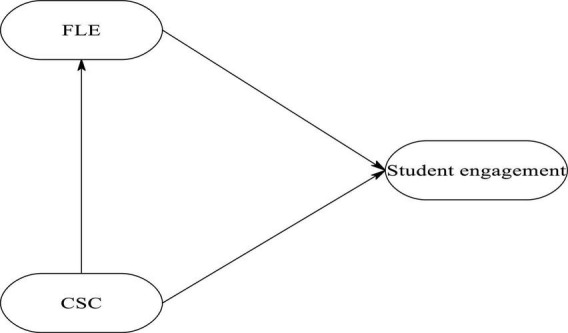
The hypothesized model.

Following Bradshaw et al. (2015) and Konold et al. (2017), it is hypothesized that CSC affects student engagement. Furthermore, classroom climate and learning contexts are argued to inculcate positive emotions in learners (Khajavy et al., 2018; Shao et al., 2019; Sampson, 2020; Li et al., 2021). As such, we added a path from CSC to FLE. The model and its hypothesized paths are shown in Figure 1.

Given the hypothesized model, the following research hypotheses were proposed in this study:

Hypothesis 1: FLE significantly predicts student engagement.

Hypothesis 2: CSC is a significant predictor of student engagement.

Hypothesis 3: CSC significantly influences FLE.

## Methodology

### Participants

For the purpose of this study, 386 intermediate Iranian EFL learners from BayaneBartar Language Center in Tehran, Iran were selected as the participants. BayaneBartar Education Center is a non-profit organization with over 250 teaching and operational staff to provide effective academic English services, namely, online and face-to-face courses for Iranian students who need English for work, study, migration, or travel. The proficiency level of the participants in this research roughly corresponded to the CEFR’s B1 or intermediate as determined by the institution’s placement test. For the sake of homogeneity and also availability, the researchers decided to select intermediate learners as the participants. Because of the ethical issues, the researchers wanted to select those learners who were willing to participate in the study. Considering willingness and agreement to participate as well as availability criteria, researchers decided to use convenience sampling (Dornyei, 2007) in this study. The sample included both male (*n* = 169) and female (*n* = 217) participants and their ages ranged from 19 to 25 (*M* = 20.88, *SD* = 1.39).

### Instruments

In order to collect the required data for this study, the following previously designed self-report scales were used. However, these scales were revalidated for the purpose and context of this study. To this end, the adequacy of the measurement models, as an approach to verify the construct validity of the scales, was examined *via* performing confirmatory factor analysis.

### Foreign language enjoyment

To assess the enjoyment of EFL learners, the scale designed by Jiang and Dewaele (2019) was used. This scale includes 10 items that were extracted from the scale originally validated by Dewaele and Macintyre (2014). The scale addresses social and private components of enjoyment. Jiang and Dewaele (2019) reported a high-reliability coefficient (α = 0.88) for this scale.

### Student engagement

The student engagement scale, validated by Reeve (2013), was used to measure participants’ engagement in this study. This scale has been designed for university students and measures four components of the construct that is: agentic engagement (AE, five items), behavioral engagement (BE, four items), cognitive engagement (CE, four items), and emotional engagement (EE, four items). Each item is measured using a seven-point Likert scale ranging from 1 (*strongly disagree*) to 7 (*strongly agree*). Reeve (2013) reported good reliability indices for all four dimensions of the scale: agentic engagement (α = 0.84), behavioral engagement (α = 0.87), cognitive engagement (α = 0.72), and emotional engagement (α = 0.91).

### Classroom social climate

Students’ perception of CSC was measured by means of the scale designed by Joe et al. (2017). The scale includes three dimensions with acceptable reliability coefficients as reported by Joe et al. (2017): Teacher academic support (TAS, three items, and α = 0.84), teacher emotional support (TES, four items, and α = 0.84), and classroom mutual respect (CMR, two items, and α = 0.71). Each item is assessed on a five-point Likert scale ranging from 1 (*strongly disagree*) to 5 (*strongly agree*).

### Procedure

Due to the COVID-19 breakout, the data were collected through an online survey. In so doing, the questionnaires for assessing the three constructs (i.e., CSC, FLE, and student engagement) were put together and converted into an online survey using the Google Docs application. Using convenience sampling procedures, the researchers requested Iranian volunteer EFL learners from the BayaneBartar Language Center to complete the online survey. The participants were encouraged to answer each item carefully. The data were gathered in the late February of 2021.

### Data analysis

The hypothesized model was tested by performing SEM. A robust multivariate procedure, SEM combines regression and factor analysis. Before running SEM, confirmatory factor analysis (CFA) was utilized to test the measurement models to confirm the construct validity of measures. Concerning the model evaluation, various goodness-of-fit indices were employed. These indices consisted of Chi-square divided by degree of freedom (χ^2^/df), comparative fit index (CFI), Tucker–Lewis Index (TLI), and root mean square error of approximation (RMSEA). According to Tseng and Schmitt (2008), a model is fit if χ^2^/df < 3, CFI and TLI > 0.90, and RMSEA < 0.08.

## Results

### Preliminary analyses

Before testing the hypothesized model of EFL student engagement, the data were screened using SPSS 22. The missing data were dealt with by using the expectation-maximization (EM) algorithm (Kline, 2011). Skewness and kurtosis indices were used to test the normality assumption of data and the values exceeding ± 2.0 were regarded as non-normal (Kunnan, 1998). Additionally, univariate and multivariate outliers were investigated by using standard scores and Mahalanobis *D*^2^, respectively (Tabachnick and Fidell, 2007). The non-normal values and outliers were excluded from analyses before running CFA and SEM. Table 1 indicates the number of valid cases for each variable.

**TABLE 1:** Number of cases for each measure.

	No of original cases	No of outliers	No of missing cases	No of valid cases
CSC	386	4	4	378
FLE	386	3	4	379
Engagement	386	5	4	377

### Construct validity of the measures

Following that, the construct validity of the measures was evaluated by running CFA. The fit indices were used to test the adequacy of the measurement models. In so doing, measurement models of CSC, FLE, and student engagement were tested. The fit indices were not initially acceptable. Consequently, some modifications were made. To this end, two items from student engagement and one item from CSC were eliminated since their factor loadings failed to exceed.40. The final models showed a good fit to the data (see Table 2). Regarding the reliability of the scales, all the calculated coefficient alphas for the scales were greater than 0.70, approving the acceptability of their internal consistencies (see Table 2). Then, descriptive statistics and correlations were calculated for all the constructs (see Table 3).

**TABLE 2:** Measurement model of the latent constructs.

	χ^2^	Df	χ^2^/df	CFI	TLI	RMSEA	Cronbach’s α
CSC	12.56	7	1.79	0.98	0.97	0.03	0.88
FLE	9.54	5	1.90	0.97	0.96	0.04	0.83
Engagement	15.78	8	1.97	0.96	0.95	0.05	0.79

**TABLE 3:** Descriptive statistics and correlations.

	*M* (*SD*)	1	2	3
(1) CSC	3.27 (0.89)	1.00		
(2) FLE	3.89 (0.91)	0.23*	1.00	
(3) Engagement	3.54 (1.02)	0.38**	0.57**	1.00

*p < 0.05. **p < 0.01.

### SEM analyses

**FIGURE 2:**
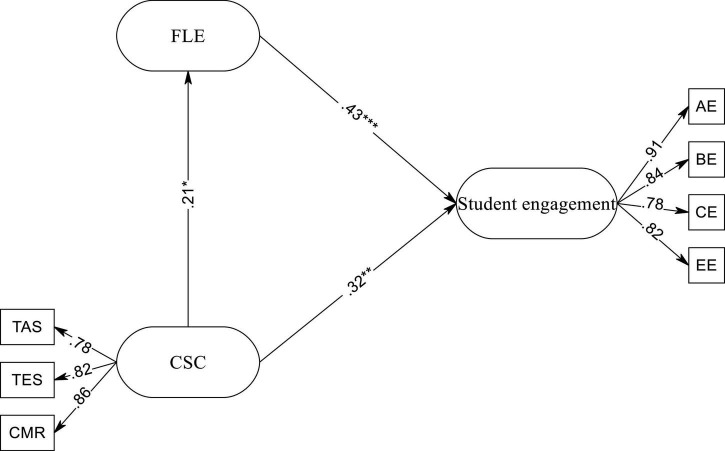
The final model of CSC, FLE, and student engagement. *p < 0.05, **p < 0.01, ***p < 0.001.

The hypothesized model was tested with AMOS with variance-covariance matrices as input and the maximum likelihood procedure. Coefficients for all paths were significant (*p* < 0.05) and the fit indices were good. Results of SEM approved all the hypotheses in the final model (see Figure 2). In order to interpret the data more meaningfully, effect size (ES) (Cohen’s *f*^2^) was calculated for all the latent variables (Table 4).

**TABLE 4:** Standardized parameter estimates for the structural model.

	*R* ^2^	*f* ^2^
(1) CSC	0.10	0.11
(2) FLE	0.18	0.21

As shown in Figure 2, CSC had a small effect on student engagement (β = 0.32, *R*^2^ = 0.10, *f*^2^ = 0.11, small effect size). Moreover, it was revealed that FLE was a stronger predictor of student engagement (β = 0.43, *R*^2^ = 0.18, *f*^2^ = 0.21, medium effect size). Also, CSC significantly predicted FLE (β = 0.21, *R*^2^ = 0.04, *f*^2^ = 0.04, small effect size).

## Discussion

This research aimed to test a structural model of student engagement based on CSC and FLE among EFL learners. SEM was used to test the associations among the latent variables. With regard to the hypothesized model and the stated hypotheses for this research, a number of significant findings were obtained.

First, it was revealed that FLE was a significant predictor of student engagement. This outcome supports those existing in the literature (e.g., Ryan and Patrick, 2001; Reeve, 2012; Dincer et al., 2019; Mercer and Dörnyei, 2020; Guo, 2021) which found that FLE is positively associated with learner engagement. From a broader perspective, this lends support to the outcomes of Pekrun and Linnenbrink-Garcia (2012), who reported that arousing pleasant emotions (e.g., FLE) can substantially affect engagement in doing learning tasks. Also, this finding, in part, supports the previous literature (Dewaele and Macintyre, 2014; Piechurska-Kuciel, 2017; Jin and Zhang, 2018; Botes et al., 2020; Li, 2020), which has documented the positive influence of FLE on academic achievement. According to the broaden-and-build theory (Fredrickson, 2001), positive emotions, such as enjoyment, can broaden learners’ thought-activity repertory and build their personal resources and affective flexibility. Positive emotions can also enhance self-discovery, acquisition of new experiences, and effective learning (MacIntyre and Gregersen, 2012; Dewaele and Macintyre, 2014; Nakamura, 2018; Wei et al., 2019; Wang Y. et al., 2021). Against this backdrop, we may argue that FLE can affect students’ engagement in learning activities by broadening their perspectives, deploying their individual resources, and enhancing their mental resilience as well as self-discovery. FLE might have increased students’ engagement by enhancing their sense of exploration and creativity (Dewaele and Macintyre, 2016). This finding partially resonates with a number of previous studies (e.g., Dewaele and Dewaele, 2017; Resnik and Schallmoser, 2019; Wei et al., 2019; Li, 2020; Zhang et al., 2020), which have evidenced the association between FLE and L2 learning outcomes.

The finding can also be justified with recourse to the control-value theory (Pekrun, 2006; Pekrun and Stephens, 2010, p. 244). More particularly, it can be argued that the link between FLE and student engagement might have been moderated by affective variables such as self-confidence, L2 motivation, and enthusiasm. Similar findings have been reported by Li et al. (2020), who found the associations between emotions and learning outcomes. Enjoyment can act as a powerful motivational drive causing academic efforts to sustain (Pekrun and Linnenbrink-Garcia, 2014). It also affects L2 self-efficacy and motivation (Kissau, 2006). From this perspective, FLE is argued to have improved EFL learners’ sustained efforts, motivation, and confidence, thereby contributing to enhancing their engagement.

Second, it was revealed that CSC predicted student engagement significantly. The positive contribution of CSC to student engagement can be justified in light of the self-determination theory, which confirms that learners might have further scholastic achievements if their sense of belonging, independence, and competence are satisfied (Connell and Wellborn, 1991). In other words, when teachers provide students with friendly and supportive learning contexts, foster interactions, and respect learners’ needs, learners are more likely to get engaged in their learning tasks (Gauley, 2017; Phung, 2017; Del Toro and Wang, 2021; Hiver et al., 2021). It seems logical that learners who have more friendly relationships with their teachers and peers are endowed with further affective and cognitive engagement in their learning process (Birch and Ladd, 1997; Hamre and Pianta, 2001).

This finding supports the bulk of existing literature that a favorable school environment is likely to enhance students’ enthusiasm, enjoyment, relatedness, and engagement (Klem and Connell, 2004; Gest et al., 2005; Gorard and See, 2011; Lee, 2012; Gietz and McIntosh, 2014). Following some studies (e.g., Rimm-Kaufman and Chiu, 2007; Mvududu and Larocque, 2008), we might argue that the pleasant school context can enhance learners’ cognitive functioning and academic performance, which, in turn, have increased their engagement in the learning process. The link between CSC and engagement of learners has been previously supported in the literature (Reyes et al., 2012; Bradshaw et al., 2015; Konold et al., 2017). Also, Konold et al. (2017) mentioned school climate as an influential construct contributing to students’ learning outcomes and their increased engagement. In other words, a pleasant, friendly, and trusting learning environment will positively affect students’ affective, cognitive, and behavioral engagement. Also, given the documented effect of student engagement on learning outcomes and academic achievement (Park, 2005; Reyes et al., 2012), this finding partially accords with the findings of several studies (e.g., Dewaele and Macintyre, 2014; Dewaele and Alfawzan, 2018; Li, 2020), which found the interconnection between FLE and L2 achievement.

Third, it was found that CSC significantly affected FLE. This outcome resonates with the findings reported by some studies (Khajavy et al., 2018; Shao et al., 2019; Sampson, 2020; Li et al., 2021; among others) in the literature, which evidenced the link between classroom environment and pleasant L2 emotions. In other words, positive learning contexts create a psychologically supportive learning environment that encourages heightened enjoyment experienced by the learners, resulting in their further exploration, sharing, and engagement (Dewaele and Macintyre, 2014; Li et al., 2021). Such a pleasant and non-threatening learning setting is also likely to decrease students’ anxiety and non-engagement in classroom activities (Barrett, 2010). Similar to the findings of Li et al. (2021), the interconnection between CSC and FLE showcases the social or environmental aspect of FLE as introduced by Dewaele and Macintyre (2014).

This finding is also in line with that of Wei et al. (2019), who reported the link between classroom environment and FLE. In other words, a favorable classroom environment contributes to enhancing FLE in EFL contexts. Similarly, De Smet et al. (2018) found that the classroom environment substantially affects learners’ emotions including enjoyment. Dewaele and Alfawzan (2018) found that FLE was a significant variable affecting language performance in the L2 contexts of Saudi Arabia and London. Likewise, Jin and Zhang (2018) reported that three dimensions of enjoyment were significantly associated with EFL achievement. It can be argued that learners who feel further enjoyment in EFL classrooms are more likely to show further involvement, concentration, persistence, interest, and strategic management in carrying out L2 tasks. Enjoyment will fuel their interest and enthusiasm in accomplishing their goals, leading to their further engagement in the learning process.

## Conclusion

In order to expand this research domain, this study sought to test a model of student engagement based on CSC and FLE among Iranian EFL learners. The SEM results showed that both CSC and FLE substantially predicted student engagement, although FLE served as a stronger predictor. Taken together, the findings of the present study support the contributions of positive psychology in L2 pedagogy (Dewaele et al., 2019), implying that pleasant perceptions of learning context and positive emotions (i.e., FLE) can lead to further student engagement. Concerning the theoretical outcomes, the outcomes of this study underscore the importance of employing positive psychology for L2 education. Given the positive effect of CSC on FLE and their joint influence on student engagement, EFL practitioners are recommended to provide their students with inviting classroom activities in supportive and favorable learning contexts. They should also establish good relations with their learners and allow their greater interactions, thereby enhancing their FLE and engagement. Treating learners kindly, caring about their concerns as well as preferences, adding fun and humor to the classroom, and allowing them to open up freely can create a comfortable and non-threatening learning setting which in turn contributes to their FLE and engagement. EFL instructors might take more practical steps to give students a pleasant experience in supportive and friendly learning environments in which more inviting topics and engaging learning tasks are provided. A variety of interesting topics and supportive contexts might enhance the FLE of students, which in turn contributes to students’ level of engagement in EFL classroom activities. The outcomes of this study give teachers a more vivid insight into the role of CSC and FLE in influencing learners’ degree of engagement. This might aid them in designing more enjoyable learning tasks to induce learners’ further participation and engagement. Teacher educators should also raise prospective teachers’ awareness of the significance of classroom climate and positive emotions in enhancing students’ engagement and learning outcomes.

This research should be viewed with respect to some limitations. First, the researchers utilized only self-report measures which are less likely to gauge learners’ actual level of CSC and FLE and their engagement. Future researchers are recommended to methodologically triangulate these findings by using qualitative elicitation techniques, such as reflective diaries and observations. Second, this study was cross-sectional and the researchers collected one wave of quantitative data. Further studies may use longitudinal designs in order to further clarify the dynamic nature of CSC, FLE, and engagement among EFL learners. Finally, the participants of this study were selected from one big language institution in Iran, thereby affecting the generalizability of the results. The generalizability of the extant findings can be enhanced by recruiting bigger samples of learners with various proficiency levels from different EFL contexts.

## Data availability statement

The data analyzed in this study is subject to the following licenses/restrictions: The raw data supporting the conclusions of this article will be made available by the authors, without undue reservation. Requests to access these datasets should be directed to corresponding author.

## Ethics statement

The studies involving human participants were reviewed and approved by the University of Kurdistan. The patients/participants provided their written informed consent to participate in this study.

## Author contributions

All authors were equally involved in designing the research, topic development, data collection, data analysis, writing drafts, and final editing.
